# A Fire and Altadena: Biographical Diffraction and Climate Disaster Loss

**DOI:** 10.1007/s11013-026-10020-5

**Published:** 2026-09-03

**Authors:** Elizabeth McKibben

**Affiliations:** Te Puna Hauora - School of Health, Victoria University of Wellington, Wellington, New Zealand

**Keywords:** Eaton fire, Autoethnography, Climate disaster, Diffraction, Loss

## Abstract

This paper develops a conceptualization of biographical diffraction via a creative autoethnography of climate disaster grief. In January 2025, the Eaton Fire devastated Altadena, California killing 19 people and destroying over 9,000 structures. Among them was my family home. Having lived abroad in Aotearoa New Zealand for 5 years, I return to Altadena after the fire, navigating loss from sites of distance and proximity. Drawing upon Barad’s concept of diffraction and the void, I grapple with the asymmetries of grief, privilege, belonging, and self that emerge with the loss of “home.” To do so, I craft a narrative with audio recordings of my footsteps walking around the debris of the Eaton Fire burn scar. By weaving together sensory vignettes, evocative writing, images, and reflections I consider how climate disaster grief is an embodied and emplaced phenomenon both historically entangled and unequally distributed. I then draw upon cross-cultural learnings to explore how Indigenous knowledge can, and cannot, inform healing of place and self from positions of settler-colonialism. Biographical diffraction is thus presented as a tool for attending to the layered emotional, material, and political dimensions of climate disaster, particularly when “home” becomes an unrecognizable site of trauma, memory, and transformation.

## Introduction

On January 7th, 2025, the Eaton Fire ignited in Altadena, California. Stoked by unprecedentedly strong winds and a late start to the wet season, the Eaton Fire lasted for 24 days, burning 14,201 acres, taking 19 lives, destroying 9,414 structures, and damaging 1,074 more (CAL FIRE, 2025). As one of the largest urban fires in California history, this event displaced more than 20,000 residents, irrevocably changing the lives of those who call Altadena home. I was raised in Altadena in our family home, where my parents still lived at the beginning of 2025. I had long left the nest and was living in Aotearoa New Zealand when the fire struck. Despite the physical distance from Altadena, this unprecedented disaster nonetheless carried shock, trauma, and grief across the world.

Diasporic experiences of climate disaster loss navigate a complex interplay of disruption and continuity. The destruction of places that shape identity, memory, and belonging leaves an indelible mark upon the psyche, wherein grief is embedded and embodied (Brinkmann, 2019). The impact of such loss is not just confined to the moments of disaster or to the first-hand experience of it. People in diaspora experience loss through a technology-mediated imagination of the event (Radeva, 2021). Phone calls, news reels, and images seen through screens enact a type of witnessing that is intimate, a-synchronous, and alters possibilities for the future. As Kuriansky (2012) explains, those in diaspora experience a vicarious trauma and “harbor thoughts of return that are dramatically disrupted by the disaster” (p. 179). Unlike residents who experienced the Eaton Fire firsthand, those in diaspora experience a continuity of their day-to-day material lives, while grieving a loss of the intangible aspects of self-concept that are linked to place.

To understand this experience of loss, I look to the concept of “biographical disruption.” Michael Bury (1982) coined this term in reference to the ways that illnesses interrupt taken-for-granted day-to-day experiences. Biographical disruption thus implies a fundamental change to one’s connection to place, community, identity, and self-concept after a particular event like a brain injury, a flood, or a fire. With such an interruption a new self-concept must emerge because of the break from emplaced and embodied habitual behaviors and daily practices which shape identity (Engman, 2019). Others extend this concept to the lives of refugees, wherein displacement forces a renegotiation of selfhood marked by abrupt and absolute change (Hart, 2023). This echoes accounts of relocation after Hurricane Katrina. After flooding uprooted communities, and forced relocation, individual’s roles within their families changed, and thus so too did their relational self-concept (Buras, 2007). Coping with biographical disruption is thus a foremost feature of climate disaster grief and loss (Gorman & Lewis, 2025).

At first glance, the Eaton Fire seems to fit the bill of a biographically disruptive event. Yet, this concept does not fully capture diasporic experiences of the fire because it privileges rupture over continuity. For those living at a distance, the disaster does not simply interrupt an existing life trajectory, it transforms an ongoing relationship with a place that shapes identity from afar. As a result, the Eaton Fire does not make a singular cut with the past, but an entanglement of continuity and disruption, wherein everyday life continues as usual at the same time as one’s sense of home is irrevocably altered. In this article, I will use my diasporic experience of the Eaton Fire to argue that disaster grief is instead better understood as a process of biographical *diffraction*.

The ‘diffraction’ I refer to in biographical diffraction is that of Karen Barad’s ethico-onto-epistemology. This way of understanding the word refutes binaries that might position continuity and discontinuity as opposites. Barad (2007) uses the physical phenomenon of diffraction, wherein light waves disperse and recombine as they encounter obstacles or apertures, to interpret social, political, and environmental relations. By emphasizing the agency of more-than-human entities, this concept is a useful metaphor for theorizing human relations with the environment, including climate disasters. Diffraction is particularly useful for understanding the multiplicity of diasporic experiences of climate disaster because, as Barad contends, “diffraction is not a singular event that happens in space and time” (Barad, 2014, p. 169). Instead, diffraction attends to patterns of dispersal that do not entail absolute separation, allowing entities to remain connected across distance. Such a framing captures the coexistence of continuity and discontinuity that characterizes diasporic experiences of climate disaster grief. Biographical diffraction, as I will argue, provides a way of understanding how life narratives, identity, and self-concept become reconfigured through dynamic relations amongst affect, place, time, and materiality of climate disaster grief.

## Methodology

This paper uses my diasporic experience of the Eaton Fire to develop the concept of biographical diffraction. Since climate disaster grief is experienced personally, but is also socially and politically produced, I use autoethnography as my method of choice. Autoethnography is a form of self-study that draws upon personal experience to reveal gritty truths about broader cultural phenomenon (Ellis et al., 2011). As an approach to inquiry, it disrupts conventional distinctions between self and culture (Reed-Danahay, 2021), unsettles boundaries between academic and evocative writing (Wilde, 2022), and can be a means of using the privilege of academic positions to make a stand against injustice in troubling times (Poulos, 2020). Via autoethnography I seek to understand the loss I experienced in the Eaton Fire as a phenomenon which reveals a pervasive socio-political ecology of erasure.

To do so requires moving beyond a purely human-centric account of climate disaster. Barad’s ethico-onto-epistemology, and corresponding diffractive methodologies, frame this erasure as emerging from the material-discursive intra-action of human and more-than-human entities. Diffractive autoethnography thus understands the self as emerging through ongoing relations with people, places, histories, and materialities. Jessica Smartt Gullion describes diffractive ethnography as “a practice of both discursive and material engagement with the world” (Gullion, 2018, 96). Following this, a diffractive *auto*ethnography, “looks at the self in entanglement with the discourses and materiality around the self” (Vu, 2018, 80). In other words, diffractive autoethnography reveals how “self” is made-with and made-differently with the discourses and materiality of everyday life. In the case of this work, “self” is made-with various entities emerging from the Eaton Fire.

In diffractive autoethnography “self” is somewhat of a fraught concept. Barad’s (2007) concept of diffraction proposes that entities are not singular or stable, calling into question the very possibility of a “self” in the first place. What diffractive autoethnographies take from this is that a notion of “self” is a mutually constitutive entity, constantly made with entities beyond “itself” (Wilde, 2020). The self, therefore, is not self-contained but a self-with: distributed across time, people, places, and things. Diffractive autoethnography is thus an ethico-onto-epistemological choice particularly suited to the study of diaspora. Because diasporic lives are already dispersed across space, the lens of diffraction allows this multiplicity to co-exist, thereby positioning climate disaster loss as an ongoing re-configuration of relationships between people, places, and material things.

To implement this approach, I draw upon techniques from sensory ethnography and creative non-fiction to construct a narrative that attends to the agency of the more-than-human world in reconfiguring self-concept. By eschewing representationalism and attending to the material world, I craft an evocative narrative with the objects and sensations that emerged from the destruction of the Eaton Fire. Four months after the fire, I travelled from Aotearoa New Zealand to Altadena, where I walked around the site of my family’s destroyed home. I captured the sound of these footsteps with a microphone attached to the hem of my jeans. Upon returning to Aotearoa New Zealand, I then listened to this recording while crafting the narrative. Repeated listening became the analytic practice through which the rhythms of walking shaped the unfolding narrative, allowing sound to function as an active participant in meaning making. Shoes, footsteps, debris, sounds, and intentionally fragmented sentences all act as diffractive agents in this story. These more-than-human relations are obstacles and apertures that redirect attention, interrupt linear meaning-making, and reveal new relations of grief, memory, self, and place in the wake of the Eaton Fire.

## Narrative

**Fig. 1:**
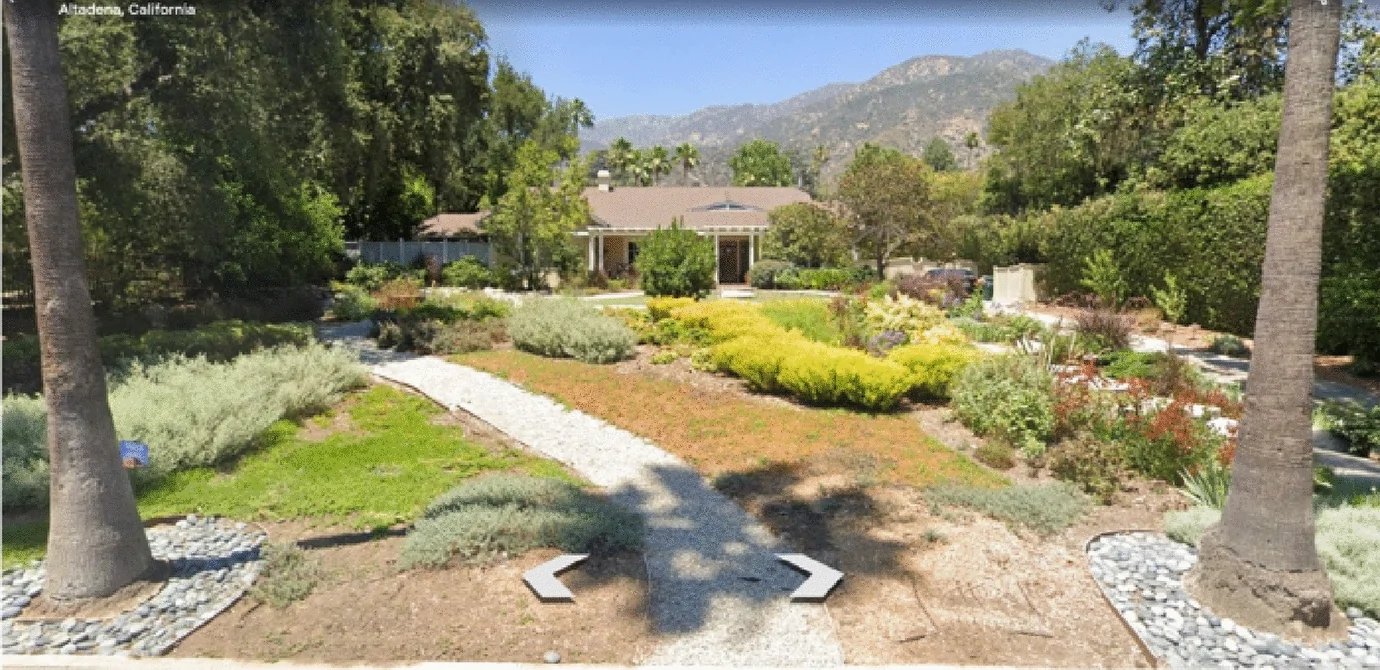
Home before the Eaton Fire, image from GoogleEarth

I once made a pair of shoes out of clay. It was a 6^th^ grade art class project teaching us about symmetry. I made sandals that looked like a face. My face. A not-quite-symmetric self-portrait. These face-shoes got packed away with other art projects, yearbooks, barbie dolls, Linkin Park CDs, and mementos of my life. They joined safely tucked memories in a box, in a closet, in a room, in a house that is gone (Fig. 1).

### Out of Step

In preparation for writing this autoethnography I flicked through the pages of Brian Fies’ (2020) graphic novel “A Fire Story.” Half-reading, half-crying. Fies (2020) mourns the loss of possessions and the memories tied to them when his home burned in Northern California.

I was in Aotearoa New Zealand when my parents called to tell me they were evacuating. I was two months away from submitting my PhD, deep in the nervousness of tying up loose ends and making sense of the last four years of my life. Mom and Dad had been giving me updates. They were safe, staying with friends. But Mom had accidentally put on mismatched shoes as they left in the darkness. I fell asleep that night optimistic that Mom’s shoes would be reunited with their pairs, and that we would be spared any further angst.

I checked my phone when I woke.

Oh God.

This just in! The Eaton Fire has moved West of Lake Street.

Oh my God.

Mandatory evacuations issued too late. At least 10 lives lost. More than 20,000 displaced. Southern California Edison power lines suspected of starting the blaze.

**Fig. 2:**
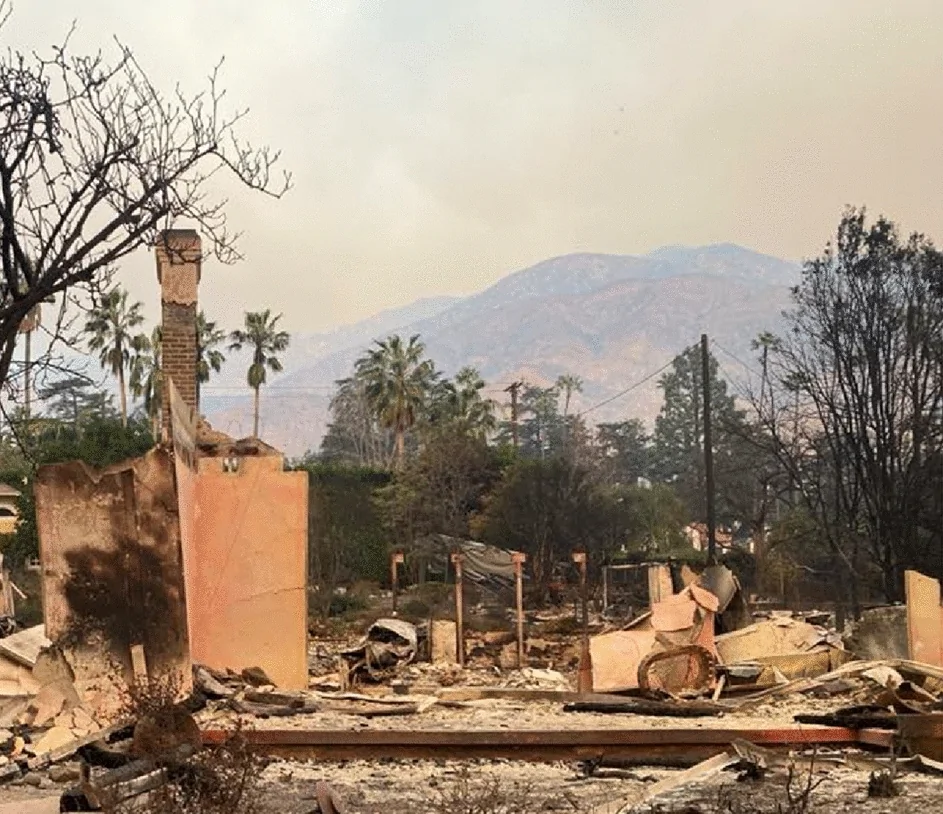
Home after the Eaton Fire

Goddamnit. When Mom called next, she broke the news. The house is gone (Fig. 2).

And now she is walking around in two mis-matched shoes that will forever remain unpaired.

I am a moth to the flame.

The news keeps showing sites from my high school running loop. The houses whose doors I knocked trick-or-treating. The middle school is up in flames. Fox’s restaurant and Everest Burger. Even the smoke shop. I cannot believe these familiar sites are engulfed in hell-scape. Maybe it’s just sensationalized. Maybe it’s just specific camera angles and none of this is real. It can’t be as bad as it looks. This has got to be fake news, right? Afterall, there is no smoke and ash and flames here, where I am, in New Zealand. It’s not real. It can’t be. Absolutely not.

That’s one of the stages of grief, right? Denial. I cannot believe it, but my body knows. Perhaps even despite the distance. A loss-induced separation of body and mind exacerbated by my physical separation from Altadena.

I am there and not-there.

In Altadena. In Aotearoa. One foot on either side of the Pacific. Here and not-here. Now and not-now. Stuck in two different time zones. Returning to the past when “home” was still a place I could securely leave my keepsakes. Then and not-then. Operating in a limbo-land of nothingness. Sleep-walking into my imagination of what went up in flames first.

The needlepoint stockings mom made us? My prom dress? The meter-tall cardboard replica of Big Ben I made in 7^th^ grade that sat in our living room for 20 years? My collection of ductaped and safety-pinned converse? Great-grandmothers piano? My Girl Scout uniforms? Those weird face-shoes? I stumble for some memory of my youth that is not burnt at the edges.

But I cannot see through the ash plume and tears. I cannot go back, not now. I have a thesis to finish! I’m about to start my first teaching role! Life is still happening here. And there’s nothing I could do about it anyway. Nothing can stop the fire. Nothing can turn back time. If I were to go home, there would be nothing there.

So, I go looking for a solidarity with home, here. *Parable of the Sower* has sat on my bookshelf unread for a year. Mom gave it to me last Christmas as a gift to remind me of home. Octavia Butler, the author, wrote from Altadena, where she is buried. This harrowing story begins with a firedream too. The protagonist, Lauren Olamina, writes in her journal,

“Fire has sprung from nowhere, has eaten in through the wall, has begun to reach toward me, reach for me...I thrash and scramble and try to swim back out of it, grabbing handfuls of air and fire, kicking, burning! Darkness.” (Butler, 1993, 4).

I hold the journaled words of a fictional child as though they are the pages of my teenage diaries, teardrops falling on the pages. I feel my story in the words of a character written from a time and place and person of Altadena that is both entirely different and exactly the same as the home I watch from afar.

Me and not-me.

Now and not-now.

There and not-there.

Here and not-here.

The Eaton Fire diffracts my life into multiple co-existing times and places. The fire keeps happening over and over and over again, in different ways, as I am unable to find my footing from afar. The fire appears again and again in the pages of *Parable of the Sowe*r. In the hunch of my shoulders as I write these words. In the questions from the guy at the convenience store, ‘You’re from LA, right? Is your family affected by the fires?’ Yes. I’m crying again. Goddamn it. It never stops. Yes. No. Even the same ole task of going to pick up a diet coke has changed.

### The Sole Falls Off

A-void.

Barad speaks on ecologies of nothingness. Of a void. Of spacetimemattering that works, “against colonialist practices of erasure and avoidance” (Barad, 2018, 56). I attempt to play with the words as they have. A-void-ance. Void-Dance. Avoidance. Barad discusses the void in the context of nuclear violence (Barad, 2018). This is not quite that. The Eaton Fire I mean. They are not equal comparisons of displacement or destruction. Yet, perhaps, there is unity in their asymmetry. It is not Hiroshima or Nagasaki or Ukraine or Gaza or Grenfell. This firestorm is not some far-off place or time that I can sympathize with ideologically from the comfort of my educated affluent white life.

It’s home.

Home (Fig. 3).

**Fig. 3:**
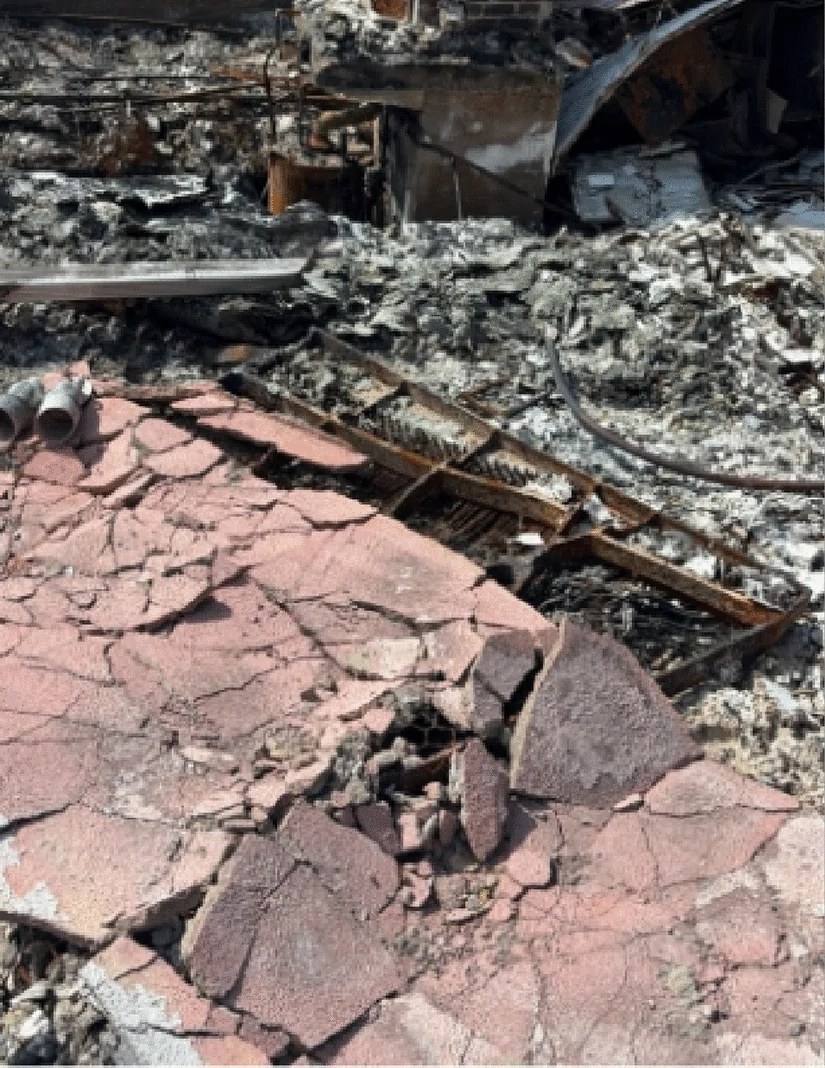
Great-grandmothers Piano

Home (Fig. 4).

**Fig. 4:**
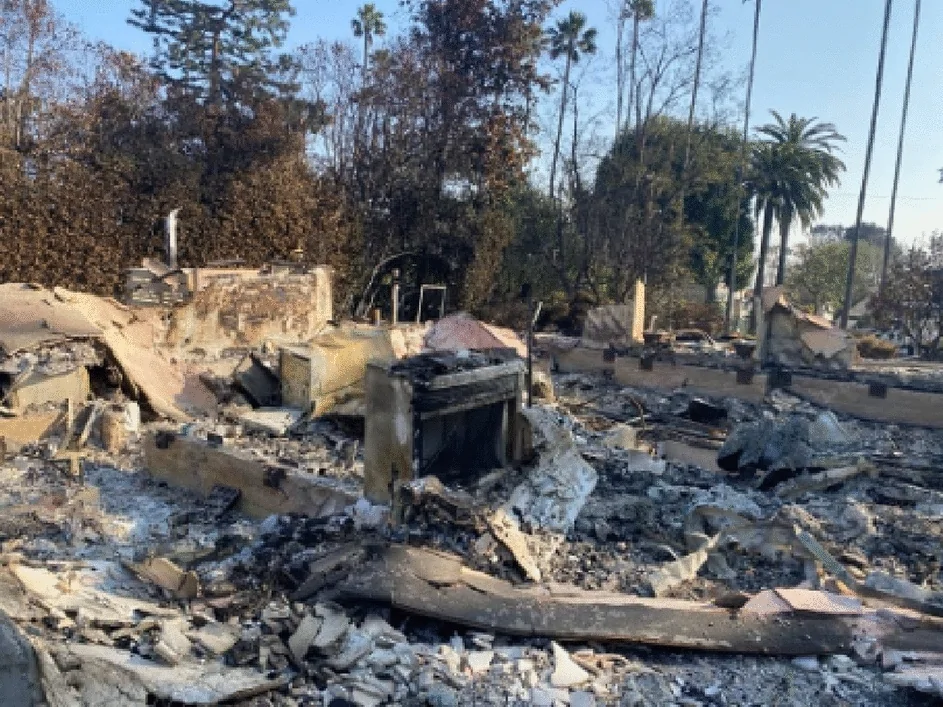
Where Christmas Stockings Hung

Home. Home. Home. Home.

I say the word so many times it begins to feel like ash in my mouth. A word that crunches under my feet. Home is now cremated bits of house getting stuck in the tread of my shoes as I walk on broken walls to make sense of this firedream.

Crunch. Crackle. Step.

Each step a friction where the sound of matter meeting matter makes something out of “nothing.” I put “nothing” in quotes because the material and sensory engagements of this diffractive autoethnography challenge the notion that “nothing” is not-thing. In contrast to Bennett’s (2010) notion of “thing-power”, here we have a “not-thing” power. This speaks to the idea that the void is not a plane of emptiness or absence, but rather infinite, and generative possibility (Barad, 2012). And in this void, there is diffractive possibility. The things and not-things of my home make a possibility for new relations to a place I thought I knew.

I keep walking forward.

One. Two. Step. Step. At least I can go “home.”

One, two, step, step. For now.

I often make jokes about intellectualizing my problems. They are half-jokes now. Going back to the debris of home with an academic orientation was a useful scaffolding for coping. A strategy for dealing with loss. A literal step-by-step guide for how I could attend to the unbelievableness of what was before me. Record the sounds. Think about the objects. Attend to the non-human entities. Listen. Feel. Think. Write. Photograph. One-two-step. What is there? What is gone? One-two-step. A way of having the nothingness speak. Of listening to the nothingness, the destruction, and noticing what appears.

Crunch. Crack. Step. Step. Everything is different.

Snap. Pop. Step. Step.

I must avoid rusting wires that popped out from...the walls? I step carefully. Tread with caution. Home has become a dizzying maze of emotions and not-things anymore.

To Barad, there is never just one path. Instead, a diffraction pattern is a “sum of all possible paths” (Barad, 2018, 66). Here, there and everywhere else. I walk around the footprint of home again. There is too much debris for my feet to leave marks. So, with each lap I find a new path. Uneven steps treading a line imperfectly and walking in un-identical circles. A listening-with-walking that folds the linearity of time. And place. I leave home to go back home.

When I return to Aotearoa, I needed to pack up my office after finishing my PhD. I brought in a suitcase to cart off my books and the sentimental chachkies I’d accumulated over the years. Postcards and trinkets and sticky notes and books, so many books, and useless crap that is now more precious to me than ever. Once full, I, in a dramatic flair, kick the suitcase lid closed.

And my shoe breaks.

The sole falls off.

The *soul* falls off.

I stare at it for a prolonged second. This shoe-less alone half-sole-soul. And then the floodgates open. I weep for weeks. And months. Trying to put out the fire with my tears. Forgetting that the fire had been extinguished months before. I still cannot believe it. My shoe breaks again and again and again and again. And I stand at the bus stop with a suitcase full of un-burnt books, leaning heavily to my left. I must now go home with uneven footsteps. Half soul-less. When I return to Aotearoa, Altadena is still alive in me. Divided selves forming one person. Before fire. After fire. Me and not-me. Two sides of a fire.

### Uneven Steps

East and West of Lake Street.

Lake Street is one of the major thoroughfares of Altadena. A straight shot to the mountains, lined with shops all the way to where it meets Altadena Drive, then becoming more residential. Now, Lake Street is also lined with debris. And Army Corps trucks. I hesitate to press my foot on the accelerator as I drive up Lake Street to get to the ruins of home.

On the east side of Lake, larger properties and early evacuation orders slowed the blaze. Many houses were spared. Many were not. That’s what money does, an extra step of protection from the firestorm. Ours was one of those houses east of Lake that was not spared. One of the doctor-rich families who to some look not-rich and to others look rich-rich. The kind of rich where the sudden cancellation of home fire insurance in 2021 was enough to swap to a less comprehensive insurance plan, but still to have one. Partially priced-out of protection.

One. Two. Step. Step.

The Palisades Fire, burning at the same time as the Eaton Fire on the other side of Los Angeles, got most of the airtime on international news. That fire, the one with the rich-rich people and celebrities filled the airwaves. What about Altadena? Altadena! WHAT ABOUT ALTADENA?! Is my home not grievable?

Judith Butler writes about what counts as a grievable life in post 9/11 US (J. Butler, 2004). Whose loss is publicly permissible? Whose loss is worth being seen and shared? Which logic of exclusion makes one fire more grievable than another? Why is Altadena seemingly more un-speakable than the Palisades?

I’m reading articles evaluating soil moisture and windpatterns and it’s all Palisades, Palisades, Palisades. With one mention of Altadena, on page 8 (Amiri et al., 2025). Where is Altadena? Where is it? Where has it gone? Home cannot just be ash and rubble. Home is people too. Where have the people gone? Dispersed now to evacuation sites, hotels, and places far away from fire.

The firestorm that is erased is the firestorm that destroyed the homes of mostly working-class people.

I tread this part of my autoethnography with caution. Uneasily. On a tightrope of the avoidance often found in performative allyship. But to ignore this would be impossible, I think. It would be one step forward and two steps back. Or perhaps not. Perhaps it is two steps forward and one step back. I listen to the recording of my footsteps. Again and again. One. Two. Step.

I had never thought much about Lake Street before, other than questioning if it would be the quickest route to take on the road to somewhere else. Nor had I ever thought much about the history of Altadena. Now, I’ve joined the Altadena Community page on Facebook, I scour through GoFundMe’s of my high school classmates, and do a deep-dive through Altadena’s Wikipedia page. Searching for a way to feel connected to home whilst I am on the other side of the world. I discover a history of Altadena that I had never needed to know before.

On the west side of Lake Street, houses were built closer together. There was more fuel for the fire. West of Lake, home to mostly Black and Brown families, now stands in complete ruin. West of Lake, evacuation orders came hours after they did for residents east of Lake. West of Lake is where all of the deaths occurred. West of Lake is now home to blocks and blocks and blocks and blocks of nothingness.

Is this why Altadena has not made the international news?

When Los Angeles’ racist red-lining policies prevented Black folks from owning homes, Altadena was one of the first places in the county where those folks could buy property. Sites of promise, where the “American Dream” is now made with smoke and mirrors. Perhaps it was always a firedream, like Octavia Butler wrote in the 80s and 90s. A firedream that can be the force of waking up. When all sides carry the load of knowing history.

I attempt to heed the advice of Māori Indigenous scholars like Moana Jackson, who have for years urged pākehā to learn our histories, to carry our part of the load of understanding the harms of colonization (Jackson, 1992). I start trying to learn about the erased history of the place I call home. What else don’t I know about Altadena?

Should I be calling it the Hahamog’na? Tongva land? Words of the Indigenous people that I have never heard spoken aloud. That I do not know how to use. Words that I did not know until I came to Aotearoa.

One. Two. Step. Step.

In 2022, a resident of Altadena returned a 1-acre lot to the Tongva Taraxat Paxaavxa Conservency (X, 2022). The first place in Los Angeles to be returned to its Indigenous people…in 200 years. It burned. A ceremonial circle has been constructed there now.

But that is not *my* story either.

One. Two. Step. Step.

The fire makes a new kind of connection. A connection to an unknown past of a familiar place. A realization that my experience of place is not stable or self-contained, it is entangled with many histories. Mine and not-mine. The firestorm has brought me to a new way of knowing myself through knowing anew.

Butler (2004) speculates, “perhaps mourning has to do with agreeing to undergo a transformation...the full result of which one cannot know in advance” (p. 21). The sound of my footsteps takes me to places I did not expect to go. To the questions about what is my story to tell. An uneven storying in which “I” am a character created with words and sounds and not-things.

I return back to the posthuman “self” for a moment, and while Judith Butler did not call it that, they certainly point out the tension of a relational self, “I am not fully known to myself because part of what I am is the enigmatic traces of others” (Butler, 2004, 46). Histories that are not mine, but nonetheless shaping my “self”. What makes *my* story? Is the owning of a story not itself a Western notion of ownership? What does that mean for not-owning?

I have been relentlessly walking forward. One. Two. Three. Four. Step. Step. Step. Step.

The fire makes a new kind of remembering.

A reminder that erasure is uneven. Asymmetric.

Hodder and Lucas (2017) ponder if there is ever symmetry in relations (Hodder & Lucas, 2017). They say, “asymmetry seems related to the positioning of a thing in relation to a larger entanglement” (Hodder & Lucas, 2017, p.126). Entanglement, then, is not a flattening of difference. Entanglement is the condition. And my rich-but-not-rich-rich whiteness is woven into a construct of identity and home that is not a stable-stagnant-solid-privileged “self”. It is woven into the larger entanglement of Altadena. Where I have never really seen West of Lake Street. And now, in this story, that asymmetry is un-a-void-able. Perhaps, the story worth telling is made by stepping back.

### Stepping Back

The nothingness ignites my ancestral curiosity.

Sometime after my visit to Altadena I go to Ireland for a conference, spending days in the National Library of Ireland looking up my last name. McKibben. And the various variations of it. MacKibbin. MacGibbon. Mac Giobúin. Walking backwards through my ancestry to a place that has not burned, and to a name that is mine, but also not. A name that has changed and been changed many times, way back when, before we were the colonizers. I try to find links between name and place. But McKibben sits scribbled on a scarce few pages of clerical records. Most of records of the places where my name lived were lost in the 1922 Irish Public Record Office…Fire.

I am of the firestorm. And it has finally caught up to me. I continue to negotiate with myself. Continue to grapple with the abstract and material nothingness that lays under my feet and in my ears as I listen again and again to the crunching of my ancestry. Does any of this really become, like Barad suggests, a “material reconfiguring of nothingness” (Barad, 2012, 16)?

There is hopefulness in learning that I have been erased before – my name changed, reconfigured, and now too perhaps, it can do so again. With/in the void lies the possibilities of knowing myself, my ancestry, and my home…differently. With/in the void lies the possibility of knowing that the loss of home is new to me, but it is not a new story.

The firestorm is etched in the footprints of the Camino Real, the pathway connecting the California missions established by the Spanish beginning in 1769. It is a firestorm kindled by my ancestors in pre-revolutionary ‘America.’ They were there, escaping their own erasure in England only to make the footprints of colonial erasure on a new land. A country in flames again. But this time, I am in the burn zone.

For the first time, to some extent, my body understands the disappearance of home. The gut-wrenching, hyperventilating, zombifying pain. The embodied grief of when home gets erased. My ideological sympathy for the harms of erasure transforms with the loss of my home. It is a type of loss that has now become embedded in my soles.

### Step. Stop. Listen

Are two feet ever symmetric? Are two shoes? They cannot ever step at the same place at the same time. Imperfect mirrors in an entangled body. And yet, they carry our gate forward. And back. And side to side. One-two-step. In a-void-dance. Making clearer the many entities that are shaped-with each other. I return to Altadena carrying what I have learned from Aotearoa, and Aotearoa walks with me here, and I carry Altadena with me to Aotearoa. A repeating dance of uncertain not-quite-reflections.

Step. Crunch. Step. Crack. Step. Stop.

When I stop trudging forward over the crunching debris of my childhood I can hear a renewal. Altadena has never been so quiet and so loud. A crow caws. And then there is birdsong I do not recall ever hearing. An owl? In the middle of the day? A robin lands just next to a fire-burnt succulent. A hummingbird flits around.

**Fig. 5:**
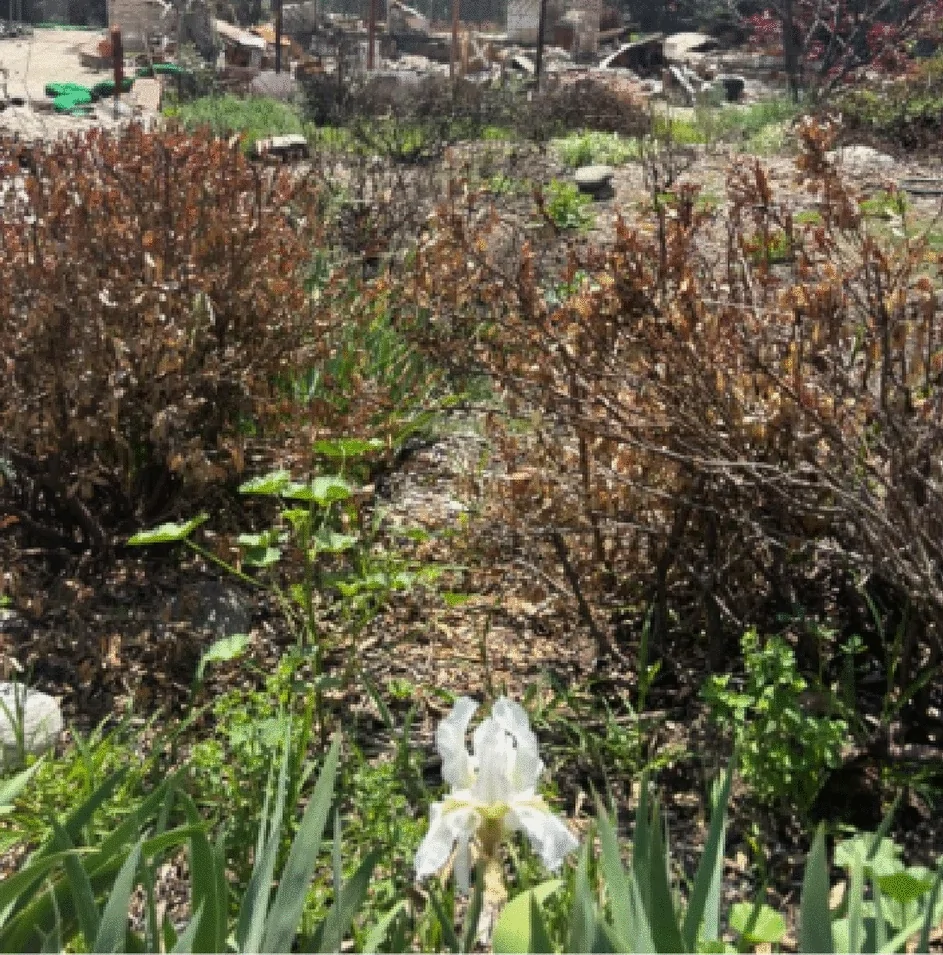
Hope is an iris

In the step-less-ness I see and hear the vitality. The hope. (Fig. 5)

Birdsong calls to me. I breath in and sing out….

Purea nei e te hau

Horoia e te ua

Whitiwhiti a e te -

The fire catches in my throat and I cannot finish Hirini Melbourne’s waiata.

What does it mean that I, an “American” sing a Māori song on Tongva land? Is it solidarity? Appropriation? Borrowing? My newest unwitting expression of white settler colonialism?

I do not think that the birds will mind if I sing to them in te reo Māori. They also wouldn’t mind if I sang in English or Spanish or Mandarin. But the point is that I sang to them waiata Māori. Seeking comfort and healing in a sound that is not my own, and that is not of this land.

This is, perhaps, my orientation to whiteness. Sarah Ahmed (2007) discusses the “body-at-home" in a phenomenology of whiteness, in particular the ways in which whiteness turns towards ‘other’ worlds in search of itself. The posthuman “self” again meets a tension with privilege. I am trying to find my loss of “body-at-home," because for the first time I do not have the feeling of being a “body-at-home." A disposed dispossessor. A posthuman tactic of seeing “self” outside of oneself, replicating a colonial grammar of taking with a gesture of reverence.

And yet, I sing. The ache in my throat demands a song. Demands something to be made in the silences of the burn scar. The choice does something. My whiteness and upper-middle classness move through me as a longing and as a loss. In my moments of stillness these identities move through me as structures that I cannot simply feel my way out of. And the singing? Well, that is a kind of diffraction too. A meeting and splintering of wounds and words across languages, geographies, and histories of fire. Literal and metaphorical. An asymmetry marked by privilege, displacement, and the mobility afforded by the very same. I am able to move between settler-colonial geographies without much question. A privileged diasporic experience.

I sing to the land with waiata Māori because I cannot recall any songs in English that fit the sole-crushing I-don’t-have-a-place-anymore pain that rings in my ears. Perhaps there are no songs like this. Or perhaps there is a song somewhere in my ancestry whose words I also do not know. Lost in a different fire.

I sing a waiata Māori about cleansing and renewal because the birds who are listening will feel me. Even though they are not kereru or kia or tui or kiwi and I am not Māori, they will feel the vibration of my heart in my throat. It is a song they know too well. And while I cannot recall a world without colonization, the land remembers what it was like before the firestorm.

With fire there is renewal. In the backyard, the half-burnt grapefruit tree thrives. Still grows. The branches are now full of foliage and little white flowers. Blossoming already. And there will be more grapefruits, the massive ones as big as my face that will fall to the ground and make popping noise like gunshots.

I begin to believe in the repair. Or a kind of repair at least. One that is cultivated from a reservoir of tiring but unexhaustive hope that I am just finding now for the first time. A hope that comes in the form of enormous grapefruits. A hope that comes in seeing Octavia’s Bookshelf offering fireaid. Of Altadena Strong murals being painted. A blossoming iris. Eyes opening.

And yet, I cannot stop weeping because Altadena has disappeared from the news. And ICE is sitting in unmarked cars along Lake Street. And the neighbors have put up signs about shooting looters. And I saw that list of banned DEI words like “Black” and “Indigenous” and “white” and “critical” and “privilege.” I reach for some memory of my country that is not burnt at the edges.

The words matter. I must say the words as often as possible so that I do not erase them too. I must say the words as often as possible while I still can. I must say the words even if I use them in not quite the right way. I must say the words so as not to forget that they are Western racial constructs that exert power-over. To take an imperfect step forward rather than a step back. Running into the fire. Even, and most especially, when the firestorm starts to seem like business-as-usual. As nothing.

### Becoming Re-soled

In *Parable of the Sower*, Lauren Olamina puts a pair of shoes in her run-away pack (Butler, 1993). A backpack of essential items ready in case of emergency. Those few core things to bring with you on a journey out of the firestorm. The possibility of a future made with protected feet and footsteps across an unimaginable landscape. The shifting landscape of before, during, and after fire. I cannot decide where “my” story fits in such a chronology. Perhaps, it is all of them. Or none. I model Olamina’s practice. Now, I have a narrative about footsteps in my metaphorical backpack. The toolkit with which I will use my position in academia to get out of the firestorm. Footsteps allow me not to avoid nothing.

I meet myself anew in an asymmetric self-portrait. Altered with footsteps walking through the here and not-here, the now and not-now. An identity that is not undone by the loss of home, but made again with the possibility of reconfiguring home with new stories, relations, and memories of place. It is not a “complete loss” as the sign stapled to the palm tree outside would suggest. Erasure is never complete (Barad, 2018). With nothingness, something opens.

Māori Indigenous scholar, Hana Tapiata writes about te Kore, a great nothingness, a faceless void that is not empty, “What’s the space in between everything? Nothing? A void? No, there’s something there. Unseen, but there” (p. 28). So, I think, there must too be something in the nothingness before me. Even if that something is just this story. Afterall, in nothingness, the fire dwindles. Nothing *can* stop the fire.

By remembering differently. By listening differently. By being differently because of being grounded in Altadena and Aotearoa.

**Fig. 6:**
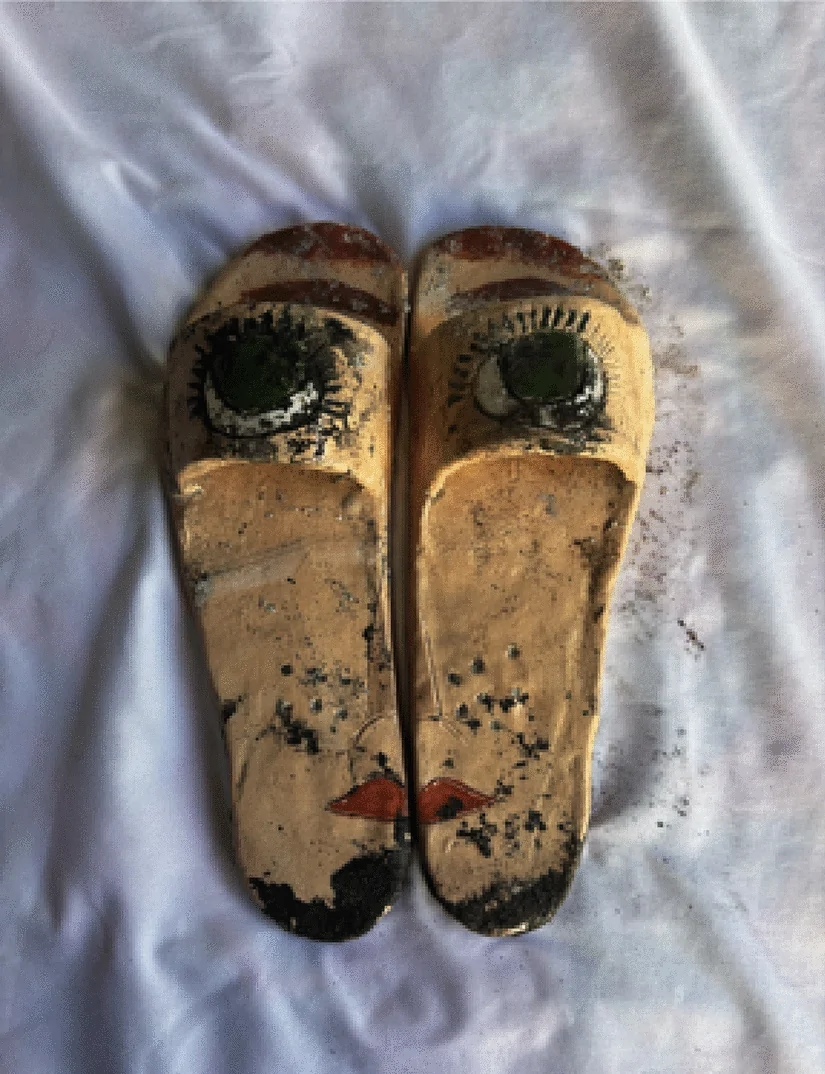
Burnt Face-Shoes

I found two unbroken items in the footprint of my destroyed home. One perfectly intact porcelain teacup. And the face-shoes (Fig. 6).

And so, I step into the future by walking into the past on the uncertain ground of home. On the grounds of being there and not-there. Me and not-me. From home I go home. With a pair of face-shoes in my backpack. With each step my self in relation to home is imagined and made again, by facing the void and remembering.

## Making Sense with Steps

As climate disasters become increasingly common, experiences of displacement and loss can no longer be understood as exceptional interruptions to an otherwise normal life. Yet, they should not be accepted as inevitable features of a changing world. This article has argued that diasporic experiences of climate disaster grief require a different conceptual vocabulary from that offered by biographical disruption. While biographical disruption foregrounds rupture from an established self-concept, the narrative presented here demonstrates that climate disaster loss simultaneously preserves and unsettles continuity of self. The destruction of home, witnessed from afar, reorients self across multiple material, spatial, temporal, political, and historical relations. This is a process that I refer to as biographical diffraction. In doing so, this article contributes both a conceptual framework for understanding diasporic climate disaster grief and a methodological demonstration of how diffractive autoethnography can generate theory through an engagement with materialities of loss. Rather than asking what happens after biography is disrupted, biographical diffraction instead enacts an ethico-onto-epistemology of self that is required for diasporic disaster grief to make sense in the first place.

Biographical diffraction, as I have called it, is a process of self-reconfiguration that attends to how identity is made-with other entities. In the case of this narrative, footsteps on rubble, a major thoroughfare, birdsong, and a mysteriously prosperous grapefruit tree re-orient relations to place. Self thus gets reconfigured in loss but is not lost entirely. Biographical diffraction therefore proposes that self is not the starting point of experience but an ongoing and emergent relation of people, places, histories, infrastructures, ecologies, and material things. Because of this, even in the face of loss, self does not break, but changes.

The narrative showcases this process via seemingly disparate encounters with material, affective, temporal, geographic, and historical relations. It begins with the face-shoes, objects that initially appear to function as a stable archive of childhood and continuity. This stability is immediately troubled with the destruction of the home in which they were meant to be kept safely. As the narrative unfolds, the narrator is simultaneously in Altadena and Aotearoa, before and after the fire, there and not-there. Mismatched evacuation shoes, a broken sole months later, repeated rhythms of footsteps, multiple sides of Lake Street, repeated fires in genealogy, and birdsong all redirect the narrative away from an autonomous autobiographical subject and towards the asymmetric relations through which self is continually enacted. The face-shoes then return altered by the fire, but ultimately unbroken. The Eaton Fire, and the destruction it caused, thus operates as a biographical aperture, a space of “nothingness” through which life is reconfigured.

Understanding the narrative in this way also explains its seemingly fragmented form. Arthur Frank (2013) describes the wounded storyteller’s chaos narratives as stories that lack order and resist neat resolution because they are lived rather than told. The narrative here shares this recursive quality, repeatedly returning to the fire through different objects, memories, and places. Selfhood does not unfold as a linear sequence with a beginning, middle, and end. Yet this movement is not senseless chaos. The recurring appearances of shoes, footsteps, streets, and walking are intra-acting motifs that iteratively redirect attention to various topics, thus forging new patterns of meaning. By reading the narrative diffractively, the story’s apparent fragmentation becomes its coherence. In other words, the internal chaos narrative makes sense of grief recursively as it is shaped by intra-related non-human entities outside of the human mind.

What ensues is a re-configuration of grief experienced at the loss of home. This grief ceases to be merely an internal psychological state, becoming a materially enacted process. Shoes, debris, technology, books, encounters at the local convenience store, and the sounds of footsteps evoke affective responses that situate loss in multiple times and places. These embodied and emplaced experiences become the means with which the “nothingness” felt in the loss of home ceases to be nothing. In the aftermath of climate disaster, absence crunches underfoot, reverberates through empty streets, and is noted in the presence of ecological liveliness. In this way, the narrative enacts a sensory-material account of grief and loss that eschews human-centricity and re-orients attention outwards in an attempt to make sense of disaster.

In this re-orientation, we see Barad’s socio-political ecology of erasure working to expand personal memory to be situated in a broader socio-political consciousness. As the story moves from the ruins of one family home to the other side of Lake Street, it encounters the pervasive history of racial segregation and the colonization of Tongva land. The Eaton Fire remains a literal and material event, but also becomes a phenomenon entangled with broader colonial, ecological, and political erasures. In attending to the material and emplaced elements of grief, the fire exposes asymmetries of race, class, and mobility that were always present but unknown. Biographical diffraction then does not flatten difference, but provides a lens through which difference becomes visible. Meanwhile, it illustrates how relations between entities are not always matching, but uneven in their entanglement with political histories of erasure. Biographical diffraction thus insists that relationality must remain attentive to asymmetry as it is informed by a multiplicity of relations to place.

In the case of this work, the diasporic movement between Altadena and Aotearoa is key in unveiling these asymmetries. The ability to move between these places, to return home, to learn from mātauranga Māori, and to produce an academic article are all products of material privilege. Rather than assume a position of ethically neutral narrator, it repeatedly returns to the tensions of white-settler positionality, leaving questions of appropriation and solidarity ultimately unresolved. The singing of *Purea Nei* remains an intentionally unsettled practice that simultaneously offers comfort drawn from one home and exposes the ongoing colonial grammars of white-settler diaspora. Rather than merely resolving grief through cultural appropriation, the encounter diffracts the loss of home across unequal histories, geographies, and relations, revealing climate disaster grief as inseparable from the colonial conditions that structure whose losses are recognized, whose movements are possible, and whose relationships to place are legitimized. Healing from the firestorm, literally and figuratively, is thus not just presented as emotional resolution, but an ongoing work of destabilizing white-settler assumptions about home, selfhood, and place which are blind to racist, classist, and colonial histories. Biographical diffraction is, perhaps, one conceptual lens which can make such work possible.

With this somewhat haphazard gathering of the scattered and ashen fragments of self-home-place-fire we find an important reminder. It is a reminder that the Eaton Fire was not just a one-time event, but a phenomenon entangled with broader colonial, ecological, and social erasures that shape the present day. With this narrative we are reminded that these erasures are almost always uneven, and it is a responsibility to remember this asymmetry by pushing past the selective amnesia of white settler memory. One that strives, in small ways, to recognize asymmetry and renegotiate privilege by remembering self and place differently. To do so is not simply a matter of prescriptive self-identification, but an ongoing process of attending to the ways in which self can become re-configured with peoples, places, things, and histories that are not of oneself.

## Data Availability

All data generated in this project, with the exception of audio recordings, are reported within the manuscript.
